# Dipeptidyl peptidase 4 (DPP-4) inhibitors and cardiovascular outcomes in patients with type 2 diabetes mellitus (T2DM): a systematic review and meta-analysis

**DOI:** 10.1186/s40360-019-0293-y

**Published:** 2019-03-04

**Authors:** Dan Liu, Biao Jin, Wei Chen, Peng Yun

**Affiliations:** 1 Department of Endocrinology, Jingzhou First Peoples Hospital, Jingzhou, Hubei People’s Republic of China; 2 Department of Critical Care Medicine, Jingzhou First Peoples Hospital, Jingzhou, Hubei People’s Republic of China

**Keywords:** Dipeptidyl peptidase 4 inhibitors, Type 2 diabetes mellitus, Cardiovascular outcomes, Cardiovascular death

## Abstract

**Background:**

Dipeptidyl peptidase 4 (DPP-4) inhibitors are newer oral anti-diabetic agents which have been approved by the Food and Drug Administration for the treatment of patients with type 2 diabetes mellitus (T2DM). In this analysis, we aimed to systematically compare the cardiovascular outcomes associated with DPP-4 inhibitors versus non-DPP-4 inhibitor users.

**Methods:**

All English publications that compared the use of DPP-4 inhibitors and that reported cardiovascular outcomes in patients with T2DM were searched using specific terms. Studies were included if they satisfied the following inclusion criteria: They were randomized trials or observation cohorts/registries comparing DPP-4 inhibitors use in patients with T2DM; The studies included a large sample size of participants; And they reported cardiovascular outcomes as their main endpoints. RevMan 5.3 was used to analyze the data, and odds ratios (OR) with 95% confidence intervals (CI) were used to represent the results.

**Results:**

A total number of 157,478 participants with T2DM were included. Seventy-six thousand and twenty six patients were assigned to the DPP-4 inhibitor group whereas 81,452 patients were assigned to the control group. Results of the current analysis showed that during a mean follow-up time period ranging from 52 to 152 weeks, the primary endpoint (cardiovascular death/non-fatal myocardial infarction (MI)/non-fatal stroke) was not significantly different in the treatment of T2DM patients with versus without DPP-4 inhibitors (OR: 0.95, 95% CI: 0.86–1.04; *P* = 0.26). Cardiovascular death (OR: 1.00, 95% CI: 0.90–1.10; *P* = 0.93), stroke (OR: 1.03, 95% CI: 0.89–1.18; *P* = 0.72), MI (OR: 0.97, 95% CI: 0.88–1.07; *P* = 0.59), all-cause mortality (OR: 0.84, 95% CI: 0.59–1.18; *P* = 0.31), hospitalization for cardiovascular complications (OR: 1.02, 95% CI: 0.96–1.09; *P* = 0.45) and hospitalization specifically for heart failure (OR: 1.05, 95% CI: 0.90–1.23; *P* = 0.55) were also similarly manifested in both groups.

**Conclusion:**

The current analysis showed that treatment with DPP-4 inhibitors did not significantly increase cardiovascular outcomes in these patients with T2DM indicating that those drugs might be safe to use in terms of cardiovascular events.

## Background

At present, even if all the patients with type 2 diabetes mellitus (T2DM) do not have the same risk, enough evidence is available regarding the occurrence of cardiovascular disease (CVD) in patients with long-standing uncontrolled T2DM [1]. Scientists are trying to develop oral hypoglycemic agents which while maintaining the blood sugar level to a constant level, could also reduce the rate of cardiovascular events.

Recently, due to the fact that oral hypoglycemic drugs while significantly maintaining a normal blood glucose level, could paradoxically increase cardiovascular events in patients with T2DM [2], the Food and Drug Administration (FDA) ordered to demonstrate their safety prior to seeking approval. Because of this reason, several newer anti-diabetic agents have undergone randomized placebo-controlled cardiovascular outcome trials (CVOT) which mainly involved patients with preexisting CVD and patients who were at a higher risk of developing this serious chronic disease [3].

Dipeptidyl peptidase 4 (DPP-4) inhibitors are newer anti-diabetic agents which have shown to well maintain blood glucose level over the long-term (decent glycated hemoglobin [HbA1c]), and were not associated with hypoglycemia or weight gain in comparison to other similar drugs [4]. However, there was a need for a systematical evidence to show the impact of DPP-4 inhibitors on cardiovascular outcomes in such patients.

In this analysis, we aimed to systematically compare the cardiovascular outcomes associated with DPP-4 inhibitors versus non-DPP-4 inhibitor users for the treatment of a large number of participants with T2DM.

## Methods

### Databases used during the search process

The search process was carried out with reference to the PRISMA guideline [5]. Medical Literature Analysis and Retrieval System Online (MEDLINE) and its interface PubMed, biomedical and pharmacological bibliographic database Excerpta Medica database (EMBASE), Cochrane database and www.ClinicalTrials.gov were searched for relevant publications.

### Search strategies and search terms

All English publications that compared the use of DPP-4 inhibitors and reported cardiovascular outcomes in patients with T2DM were searched specifically using the terms: “dipeptidyl peptidase 4 inhibitors and type 2 diabetes mellitus”, “dipeptidyl peptidase 4 inhibitors and diabetes mellitus and cardiovascular outcomes”, “dipeptidyl peptidase 4 inhibitors and cardiovascular outcomes”, “dipeptidyl peptidase 4 inhibitors and cardiac”, “DPP-4 inhibitors and diabetes mellitus”.

In addition, individual name of the drugs were also used: “sitagliptin and type 2 diabetes mellitus”, “sitagliptin and cardiovascular outcomes”, “sitagliptin and diabetes mellitus and cardiovascular outcomes”, “saxagliptin and type 2 diabetes mellitus”, “saxagliptin and cardiovascular outcomes”, “saxagliptin and diabetes mellitus and cardiovascular outcomes”, “omarigliptin and type 2 diabetes mellitus”, “omarigliptin and cardiovascular outcomes”, “omarigliptin and diabetes mellitus and cardiovascular outcomes”, “alogliptin and type 2 diabetes mellitus”, “alogliptin and cardiovascular outcomes”, “alogliptin and diabetes mellitus and cardiovascular outcomes”, “linagliptin and cardiovascular outcomes”, “linagliptin and diabetes mellitus and cardiovascular outcomes”, “vildagliptin and cardiovascular outcomes”, “vildagliptin and diabetes mellitus and cardiovascular outcomes”.

### Inclusion and exclusion criteria

Studies were included if they satisfied the following inclusion criteria:

- They were randomized trials or observation cohorts/registries comparing DPP-4 inhibitors use in patients with T2DM;
- They included a large sample size of participants (note that studies with very small sample size were excluded);
- They reported cardiovascular outcomes as their main endpoints;

Studies were excluded if they consisted of the following features:

- They were literature reviews/meta-analyzes/case studies/letters to editors;
- They did not include DPP-4 inhibitor users for the treatment of patients with T2DM;
- They included a small sample size;
- They did not report cardiovascular outcomes as their endpoints;
- They were written in other languages than in English;
- They were duplicated studies.

### Type of DPP-4 inhibitors, cardiovascular outcomes reported and follow-up time periods

Omarigliptin, sitagliptin, saxagliptin and alogliptin were the DPP-4 inhibitors which were used to treat these patients with T2DM as shown in Table 1.

**Table 1:** Type of DPP-4 inhibitors, cardiovascular outcomes reported and follow-up time periods

Studies	Type of DPP-4 inhibitors	Cardiovascular outcomes reported	Mean follow-up time period
Gantz2017 [7]	Omarigliptin	Cardiovascular death/non-fatal MI or non-fatal stroke, cardiovascular related death, fatal and non-fatal MI, fata and non-fatal stroke, all-cause mortality, hospitalization for heart failure, hospitalization for heart failure or cardiovascular death	96 weeks
Green2015 [8]	Sitagliptin	Cardiovascular death/non-fatal MI or non-fatal stroke, cardiovascular death, non-fatal MI, non-fatal stroke, hospitalization for unstable angina, hospitalization for heart failure, all-cause mortality	152 weeks
Park2015 [9]	Unspecified DPP-4 inhibitors	All-cause mortality	124 weeks
Scirica2013 [10]	Saxagliptin	Cardiovascular death/non-fatal MI or non-fatal stroke, all-cause mortality, cardiovascular death, MI, stroke, hospitalization for unstable angina, hospitalization for heart failure, hospitalization for coronary revascularization	109 weeks
Shih2016 [11]	Unspecified DPP-4 inhibitors	All-cause mortality, MACEs, MI, stroke, heart failure	114 weeks
Wang2015 [12]	Sitagliptin	Cardiovascular death/non-fatal MI or non-fatal stroke, MI, stroke, cardiovascular mortality	52 weeks
White2013 [13]	Alogliptin	Cardiovascular death/non-fatal MI or non-fatal stroke, cardiovascular mortality, non-fatal MI, non-fatal stroke, all-cause mortality	78 weeks

Abbreviations: *DDP-4* Dipeptidyl peptidase 4, *MI* Myocardial infarction, *MACEs* Major adverse cardiac events

Primary endpoint: including cardiovascular death/non-fatal MI or non-fatal stroke, cardiovascular related death, fatal and non-fatal MI

The following outcomes were assessed:

- Primary endpoint: consisting of cardiovascular death, non-fatal myocardial infarction and non-fatal stroke;
- Cardiovascular death;
- Myocardial infarction (MI);
- Stroke;
- All-cause mortality;
- Hospitalization for cardiovascular complications;
- Hospitalization specifically for heart failure.

A mean follow-up time period ranging from 52 weeks to 152 weeks were considered relevant as shown in Table 1.

### Data extraction and quality assessment

Four independent reviewers were responsible for the data extraction and quality assessment of the trials. In the beginning, each reviewer extracted the following data: the names of the authors, the year of publication, the type of DPP-4 inhibitors, the cardiovascular outcomes, the average follow-up time periods, the total number of participants from each group, the baseline features, the duration of diabetes mellitus, the total number of cardiovascular events; and at a later stage, data were compared and cross-checked to make sure all correct data were entered.

Quality assessment of the trials was carried out with reference to the criteria suggested by the Cochrane Collaboration [6]. A maximum total score of 12 points was allotted based on the bias risk reported.

### Statistical analysis

RevMan 5.3 software was used to carry out the statistical analysis of the pooled data. Odd ratios (OR) and 95% confidence intervals (CI) were generated to represent the main analytical data throughout the result section.

Expected heterogeneity was assessed using the (1) Q statistic test whereby a result with a *P* value less or equal to 0.05 was considered statistically significant, and (2) the I^2^ statistic test whereby a lower I^2^ value denoted a lower heterogeneity.

A fixed statistical effect model (I^2^ < 50%) or a random statistical effect model (I^2^ > 50%) was applied depending upon the value of heterogeneity which was generated.

Sensitivity analysis was also carried out to compare with the main results for any significant difference by a method of exclusion.

Since this analysis included only a very small volume of studies, publication bias was visually assessed through funnel plots which were generated through the RevMan software.

### Compliance with ethical guidelines

This is a systematic review and meta-analysis of previously published original studies and therefore ethical approval or any board review approval was not required.

## Results

### Search outcomes

Electronic search resulted in a total number of 4512 publications. An initial assessment was carried out to eliminate unwanted studies, and based on relevance, only 245 full-texts were finally assessed for eligibility.

After another round of assessment, further eliminations were carried out based on the following criteria:

- Literature review/meta-analyses/case studies/letters to editors (*n* = 32);
- Cardiovascular outcomes were not reported (*n* = 22);
- Consisted of a small number of participants (*n* = 49);
- Did not report the correct control group (n = 22);
- Duplicates (*n* = 113).

**Fig. 1:**
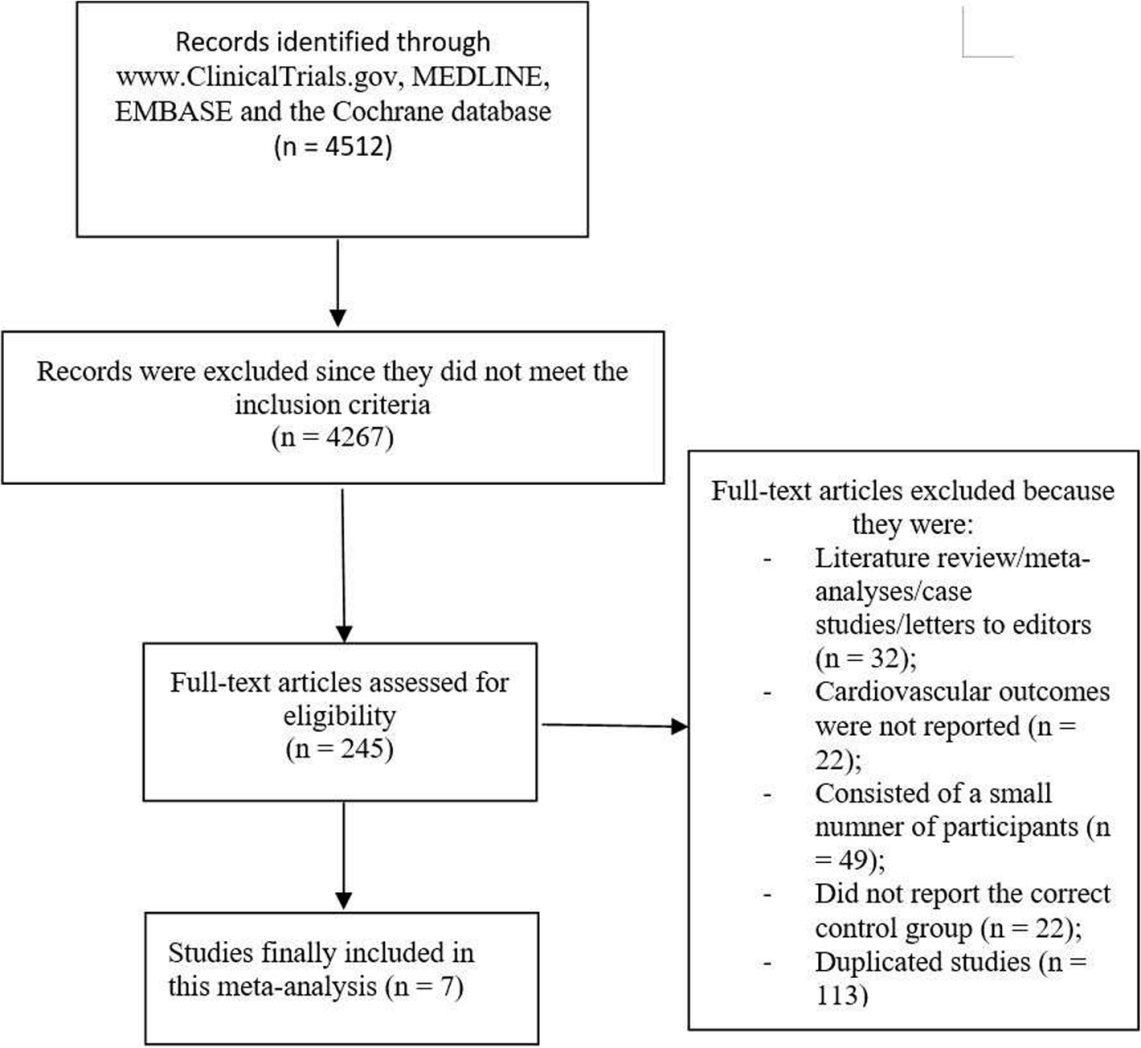
Flow diagram showing the study selection

Finally, only 7 studies (4 randomized controlled trials and 3 observational cohorts) [7–13] were selected for this analysis as shown in Fig. 1.

### Main features of the studies

Four studies were randomized controlled trials and 3 studies were observational cohorts.

A total number of 157, 478 participants with T2DM were included in this analysis. Seventy-six thousand and twenty six (76, 026) patients were assigned to the DPP-4 inhibitor group whereas 81, 452 patients were assigned to the control group as shown in Table 2. Patients were enrolled between the years 2007 to 2017 as shown in the Table.

**Table 2:** Main features of the studies

Studies	Type of study	Time period of patients’ enrollment	Total no of patients assigned to DPP-4 inhibitors (n)	Total no of patients assigned to control group (n)
Gantz2017 [7]	RCT	2012–2017	2092	2100
Green2015 [8]	RCT	2008–2015	7332	7339
Park2015 [9]	OS	2007–2011	1866	5179
Scirica2013 [10]	RCT	2010–2011	8280	8212
Shih2016 [11]	OS	2009–2013	53,208	53,208
Wang2015 [12]	OS	2009–2011	547	2735
White2013 [13]	RCT	2009–2013	2701	2679
Total no of patients (n)			76,026	81,452

Abbreviations: *RCT* Randomized controlled trials, *OS* Observational studies, *DPP-4* dipeptidyl peptidase 4

Based on the methodological assessment, a score ranging from 8 to 12 were allotted to the trials indicating a low to moderate risk of bias.

### Baseline features of the participants

The baseline features of the participants have been listed in Table 3.

**Table 3:** Baseline features of the participants

Studies	Age (years)	Males (%)	Duration of DM (years)	HbA1c (%)	HBP (%)	CS (%)
	DP/NDP	DP/NDP	DP/NDP	DP/NDP	DP/NDP	DP/NDP
Gantz2017	63.7/63.6	69.6/70.7	12.0/12.1	8.00/8.00	95.1/95.6	14.3/14.5
Green2015	–	–	–	–	–	–
Park2015	61.0/63.0	66.2/62.2	–	7.60/7.20	79.7/80.4	–
Scirica2013	65.1/65.0	66.6/67.3	10.3/10.3	8.00/8.00	81.2/82.4	–
Shih2016	74.5/74.5	46.2/46.2	8.70/8.70	–	90.9/90.9	–
Wang2015	66.0/65.9	64.0/63.7	–	–	75.1/75.9	–
White2013	61.0/61.0	67.7/68.0	7.10/7.30	8.00/8.00	82.5/83.6	13.0/14.3

Abbreviations: *DM* Diabetes mellitus, *HbA1c* Glycated hemoglobin, *HBP* High blood pressure, *CS* Current smoker, *DP* Dipeptidyl peptidase 4 inhibitor group, *NDP* Non-dipeptidyl peptidase 4 inhibitor group

As shown in the Table, a mean age of 61.0–74.5 years were reported among the participants. Most of the participants were male patients with a mean percentage of 46.2–70.7%. The duration of diabetes mellitus varied from 7.10 to 12.1 years. The participants had an average HbA1c varying from 7.20 to 8.00 years. Other features which were reported in Table 3 included the percentage of participants with hypertension and current smoker.

### Analysis of the cardiovascular outcomes

**Fig. 2:**
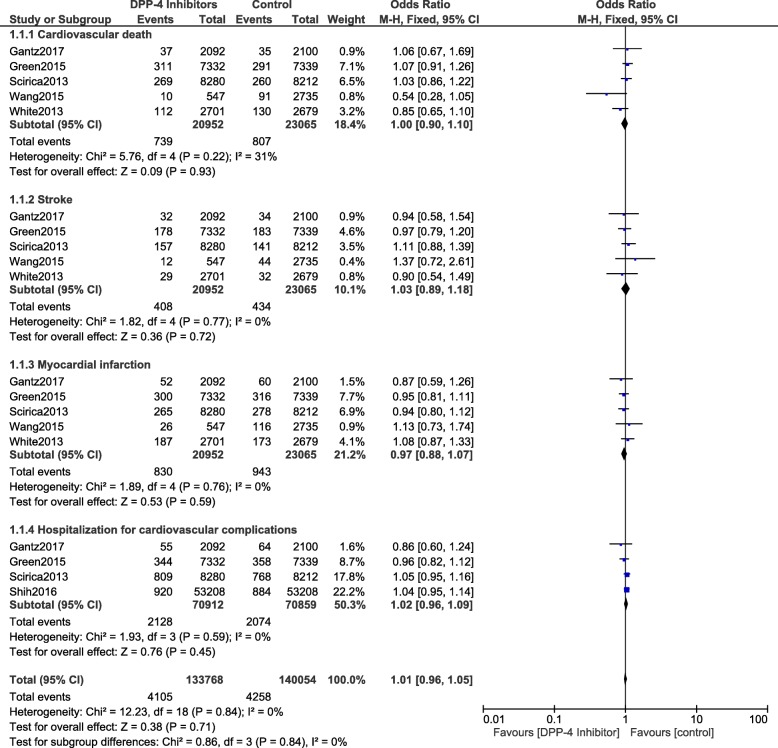
Cardiovascular outcomes observed with DPP-4 inhibitor users versus the control group (part 1)

**Fig. 3:**
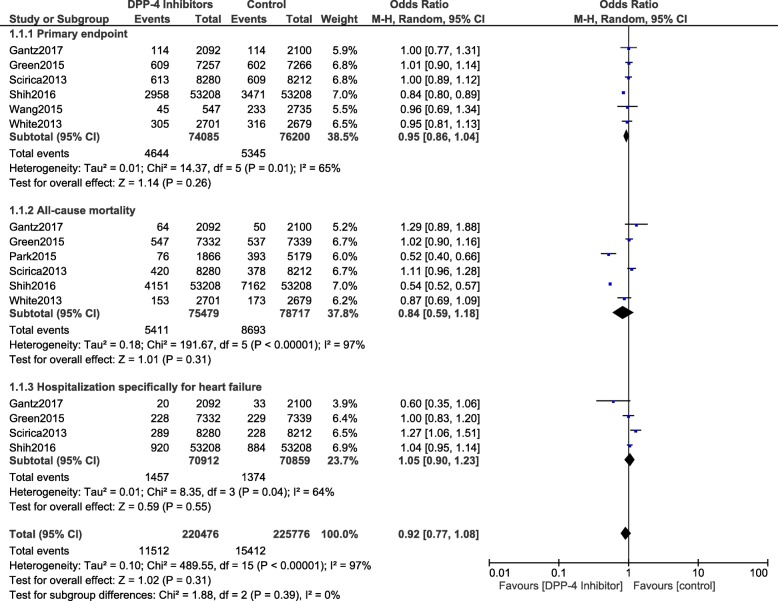
Cardiovascular outcomes observed with DPP-4 inhibitor users versus the control group (part 2)

The current analysis showed that during a mean follow-up time period ranging from 52 weeks to 152 weeks, the primary endpoint was not significantly different in the treatment of T2DM patients with versus without DPP-4 inhibitors (OR: 0.95, 95% CI: 0.86–1.04; *P* = 0.26). Cardiovascular death (OR: 1.00, 95% CI: 0.90–1.10; *P* = 0.93), stroke (OR: 1.03, 95% CI: 0.89–1.18; *P* = 0.72), MI (OR: 0.97, 95% CI: 0.88–1.07; *P* = 0.59), all-cause mortality (OR: 0.84, 95% CI: 0.59–1.18; *P* = 0.31), hospitalization for cardiovascular complications (OR: 1.02, 95% CI: 0.96–1.09; *P* = 0.45) and hospitalization specifically for heart failure (OR: 1.05, 95% CI: 0.90–1.23; *P* = 0.55) were also similarly manifested in both groups. The results have been represented in Figs. 2 and 3.

The main results have also been summarized in Table 4.

**Table 4:** Results of this analysis with a large population size

Cardiovascular outcomes assessed	No of studies involved (n)	OR with 95% CI	P value	I^2^ value (%)
Primary endpoint	6	0.95 [0.86–1.04]	0.26	65
Cardiovascular death	5	1.00 [0.90–1.10]	0.93	31
All-cause mortality	6	0.84 [0.59–1.18]	0.31	97
Stroke	5	1.03 [0.89–1.18]	0.72	0
Myocardial infarction	5	0.97 [0.88–1.07]	0.59	0
Hospitalization for cardiovascular complications	4	1.02 [0.96–1.09]	0.45	0
Hospitalization specifically for heart failure	4	1.05 [0.90–1.23]	0.55	64

Abbreviations: *OR* Odds ratios, *CI* Confidence intervals

### Sensitivity analyses and publication bias

Sensitivity analysis was carried out for the respective subgroups and consistent results were obtained throughout. When study Gantz2017 was excluded, results for primary endpoint (OR: 0.94, 95% CI: 0.85–1.04; *P* = 0.25), cardiovascular death (OR: 0.99, 95% CI: 0.89–1.10; *P* = 0.88), stroke (OR: 1.03, 95% CI: 0.89–1.19; *P* = 0.66), MI (OR: 0.98, 95% CI: 0.89–1.09; *P* = 0.73), hospitalization for cardiovascular complications (OR: 1.03, 95% CI: 0.97–1.10; *P* = 0.36), all-cause mortality (OR: 0.78, 95% CI: 0.53–1.12; *P* = 0.18) and hospitalization specifically for heart failure (OR: 1.09, 95% CI: 0.96–1.23; *P* = 0.20) were not significantly different compared to the main analysis. Consistent results were obtained throughout.

When study Green2015 was excluded, results for primary endpoint (OR: 0.93, 95% CI: 0.84–1.02; *P* = 0.14), cardiovascular death (OR: 0.95, 95% CI: 0.83–1.08; *P* = 0.42), stroke (OR: 1.07, 95% CI: 0.89–1.28; *P* = 0.48), MI (OR: 0.99, 95% CI: 0.88–1.12; *P* = 0.86), hospitalization for cardiovascular complications (OR: 1.04, 95% CI: 0.97–1.11; *P* = 0.28), all-cause mortality (OR: 0.80, 95% CI: 0.55–1.18; *P* = 0.26) and hospitalization specifically for heart failure (OR: 1.05, 95% CI: 0.83–1.32; *P* = 0.69) were not significantly different compared to the main analysis. Consistent results were obtained throughout.

Even when study Shih2016, which was the largest study (with the highest number of participants) in this analysis, when excluded, results for the primary endpoint (OR: 0.99, 95% CI: 0.93–1.07; *P* = 0.87), all-cause mortality (OR: 0.92, 95% CI: 0.72–1.17; P = 0.48), hospitalization specifically for heart failure (OR: 1.01, 95% CI: 0.76–1.35; *P* = 0.95), and hospitalization for cardiovascular complications (OR: 1.01, 95% CI: 0.93–1.10; *P* = 0.80) were not significantly different compared to the main results of this current analysis, that is, still consistent results were obtained throughout.

The same results were obtained even when the remaining studies were excluded by turn and new analyses were carried out.

**Fig. 4:**
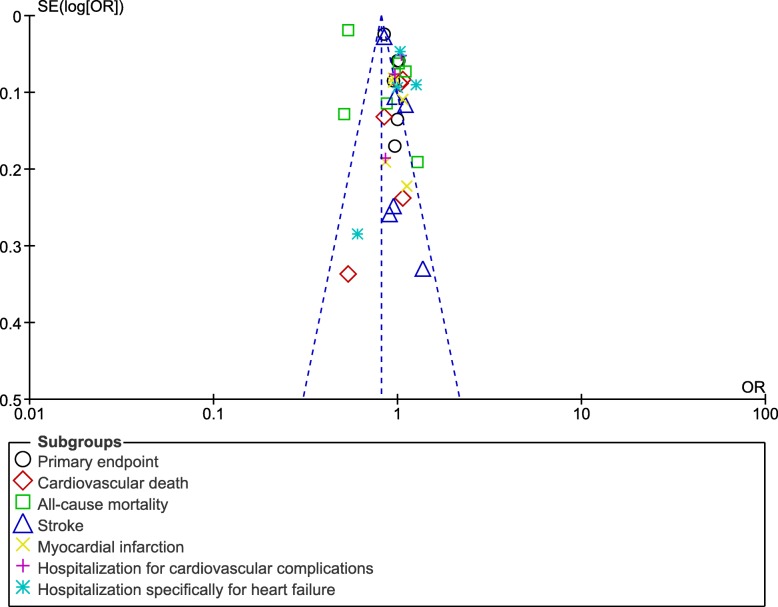
Funnel plot representing publication bias

Also, a low evidence of publication bias was observed throughout, across all the trials and observational cohorts that assessed the cardiovascular outcomes between the DPP-4 inhibitor versus the non-DPP-4 inhibitor group as shown in Fig. 4.

## Discussion

DPP-4 inhibitors are newer oral anti-diabetic agents with high expectations. Their mechanism of action is based on the prolongation of the activity of glucagon-like peptide 1 (GLP1), the gastric-inhibitory peptide, as well as other incretins by restraining their breakdown [14].

The current analysis which included a very large total number of participants showed that DPP-4 inhibitors were not associated with significantly higher cardiovascular outcomes in comparison to DDP-4 inhibitor non-users. Cardiovascular death, MI, hospitalization for cardiovascular complications and specifically for heart failure, were similarly manifested with DPP-4 inhibitors in these patients with T2DM.

The SAVOR-TIMI 53 trial [10] which was a randomized, multicenter, double blind placebo-controlled phase 4 trial which demonstrated the cardiovascular safety and efficacy of DPP-4 inhibitors also did not show any increase in cardiovascular events associated with the use of this group of drugs. However, an increased rate of hospitalization due to heart failure was observed.

In the EXAMINE trial [13], whereby 5380 participants underwent randomization, the authors concluded that no increase in adverse cardiovascular events were observed in those patients who were recently affected by acute coronary syndrome.

Even in the TECOS trial [8], which was also a randomized, double-blind study involving more than 10, 000 participants, the authors did not observe any significant increase in cardiovascular outcomes with the use of DPP-4 inhibitors for the treatment of patients with T2DM. However, compared to the SAVOR-TIMI 53 trial, there was no increase in hospitalization due to heart failure in the TECOS trial.

Another open observational non-crossover retrospective cohort study which was conducted between June 2012 and December 2013, and which compared the cardiovascular efficacy and safety of linagliptin, another DPP-4 inhibitor, also did not show any significant increase in cardiovascular events with the drug in these patients with T2DM and acute coronary syndrome [15]. However, the forthcoming CARMELINA trial which aimed to demonstrate the effects of linagliptin on cardiovascular and renal outcomes might further add information to DPP-4 inhibitors and cardiovascular events [16].

### Limitations

First of all, the inclusion of data which were extracted from observational studies have increased the heterogeneity during subgroup analysis. This might be one major limitation of this analysis. Secondly, the duration of T2DM was not similar in all the studies. In addition, the follow-up time periods were different in different studies, and this might have affected the results. Also, different DPP-4 inhibitors were combined prior to analysis and this might have also contributed to the limitations observed in this analysis.

## Conclusions

The current analysis showed that treatment with DPP-4 inhibitors did not significantly increase cardiovascular outcomes in these patients with T2DM indicating that those drugs might be safe to use in terms of cardiovascular events.

## Abbreviations

CVD: Cardiovascular diseases
DPP-4: Dipeptidyl peptidase 4
OR: Odds ratios
T2DM: Type 2 diabetes mellitus
